# Combination Drug Delivery Approaches in Metastatic Breast Cancer

**DOI:** 10.1155/2012/915375

**Published:** 2012-04-26

**Authors:** Jun H. Lee, Anjan Nan

**Affiliations:** ^1^Department of Pharmaceutical Sciences, School of Pharmacy, University of Maryland at Baltimore, Baltimore, MD 21201, USA; ^2^University of Maryland Marlene and Stewart Greenebaum Cancer Center, Baltimore, MD 21201, USA

## Abstract

Disseminated metastatic breast cancer needs aggressive treatment due to its reduced response to anticancer treatment and hence low survival and quality of life. Although in theory a combination drug therapy has advantages over single-agent therapy, no appreciable survival enhancement is generally reported whereas increased toxicity is frequently seen in combination treatment especially in chemotherapy. Currently used combination treatments in metastatic breast cancer will be discussed with their challenges leading to the introduction of novel combination anticancer drug delivery systems that aim to overcome these challenges. Widely studied drug delivery systems such as liposomes, dendrimers, polymeric nanoparticles, and water-soluble polymers can concurrently carry multiple anticancer drugs in one platform. These carriers can provide improved target specificity achieved by passive and/or active targeting mechanisms.

## 1. Introduction

Breast cancer is the most common cancer in females and the second most common cause of death in women in the United States [1]. Metastatic breast cancer is the most-advanced stage of breast cancer involving the dissemination of cancerous cells from the breast to other areas of the body. At the time of diagnosis, less than 10% of women are presented with a metastatic disease. However, when relapse occurs after definitive therapy for early stage or locally advanced disease, the majority of patients end up with disseminated metastases rather than an isolated local recurrence. The median survival for metastatic breast cancer patients appears to have improved over time, which has been attributed to the availability of new more effective agents, including taxanes, aromatase inhibitors, and anti-HER2 agents [2, 3]. However, metastatic breast cancer is unlikely to be completely cured and the survival is extremely low as five-year survival is attained in only 23.4% of these patients. Therefore it is important to understand the patients' treatment goals and the need for aggressive therapy including combination therapy [4]. The primary goals of systemic treatment of metastatic breast cancer are prolonged survival, alleviated symptoms, and maintained or improved quality of life despite the toxicity associated with treatment [5–8]. Although combining chemotherapy, biologic therapy, and/or endocrine therapy might have additive and even synergistic efficacy in theory, it generally leads to increased toxicity. Clinical trials have failed to show a clear survival advantage for the concurrent administration of chemotherapy and endocrine therapy over either single modality [5, 9, 10]. Novel biologic therapies, that specifically target molecular pathways, such as angiogenesis (growth of new blood vessels from preexisting vessels especially around tumors) and other growth factors relevant to the development of breast cancer, have contributed to advancing the treatment and improving the prognosis of metastatic breast cancer. Noncytotoxic biological agents act on specific molecular pathways to target cancer cells while sparing normal tissues, and therefore do not generally cause alopecia, vomiting, and myelosuppression that are characteristic of cytotoxic drugs. New agents and combination regimens clearly have the potential to significantly improve clinical outcomes, yet they also create new challenges including limited patient population, tolerability, and compliance issues [11]. Over the last decade, carrier-mediated drug delivery systems such as liposomes, dendrimers, nanoparticles, water-soluble polymer-drug, and polymer-protein conjugates have emerged as a new class of antitumor agents [12–14]. The advantages of carrier-mediated drug delivery over conventional anticancer therapy include: (1) passive tumor targeting due to the enhanced permeability and retention (EPR) effect [15], (2) active targeting by additionally introducing receptor specific ligands to the carriers [12], (3) lower toxicity of bound or encapsulated drug [16], and (4) intracellular endocytotic uptake with the potential to bypass mechanisms of drug resistance, including p-glycoprotein-mediated multidrug efflux [13]. Drug delivery systems derived from liposomes, dendimers, polymeric nanoparticles and micelles are currently under preclinical and clinical development as novel nanomedicines that can deliver a combination of multiple drugs to various cancers. The present paper highlights the currently available combination therapy approaches including emerging novel carrier-mediated drug delivery systems with an emphasis on metastatic breast cancer.

## 2. Combination Therapy in Metastatic Breast Cancer

**Figure 1:**
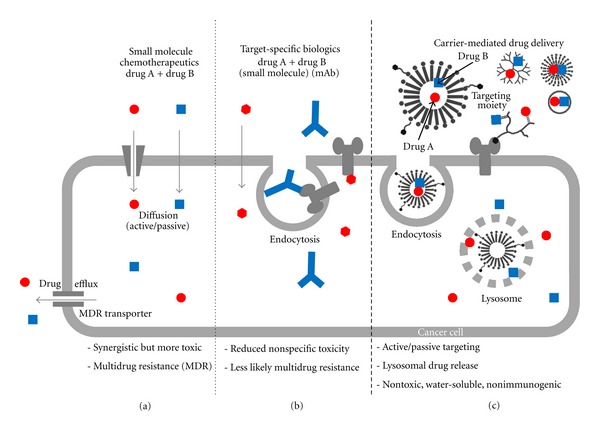
Schematic representation of various combination drug delivery approaches for treatment of cancer. (a) combination of small molecule chemotherapeutic agents, (b) combination of target specific biologic agents including monoclonal antibodies, and small molecule chemotherapeutics (c) carrier-mediated combination drug delivery (see Figures 2 to 5 for structures of various carriers).

For better therapeutic effectiveness combination anticancer treatment has long been adopted in clinics. The general rationale for employing combination therapy is twofold. First, when multiple drugs with different molecular targets are applied, the cancer adaptation process such as cancer cell mutations can be delayed. Second, when multiple drugs target the same cellular pathway they could function synergistically for higher therapeutic efficacy and higher target selectivity. Currently available combination regimens for metastatic breast cancer in clinics are limited to administrating a physical mixture of two or more anticancer agents. The clinically used combination regimens in the US can be broadly classified based on their mechanisms of action (Figures 1(a) and 1(b)) including: (1) combination of nonspecific small molecule chemotherapeutic agents, (2) combination of target-specific biologic agent and small molecule chemotherapeutic agents, and (3) combination of target-specific biologic agents.

### 2.1. Combination of Nonspecific Small Molecule Chemotherapeutic Agents

Small molecule chemotherapeutic agents can be given singly or in combination (Figure 1(a)). Toxicity is typically less with single-agent therapy and quality of life appears better. However, combination therapy may be a more appropriate first-line choice for symptomatic patients or those with rapidly progressive visceral metastases because of the greater likelihood of an objective response. Of the many active combination chemotherapy regimens in metastatic breast cancer (Table 1), none is established as the optimal first-line regimen. For example prior exposure to anthracyclines and/or taxanes is a major limiting factor when selecting such a regimen since it often renders tumors resistant and is therefore related to reduced clinical benefits including response rate upon rechallenging to these chemotherapeutic classes and even to other classes of drugs [17, 18].

#### 2.1.1. Anthracycline-Based Regimens

With response rates of up to 60% in previously untreated patients with metastatic breast cancer anthracycline-based regimens are one of the most widely used first-line chemotherapies. Because of this advantage patients relapsing more than 12 months after anthracycline-based treatment may be reinduced with anthracycline-based combination chemotherapy [19]. Anthracyclines (or anthracycline antibiotics), derived from *Streptomyces* bacteria, are a class of drugs widely used and studied in cancer chemotherapy. Mechanisms of action of anthracyclines are (1) to inhibit DNA and RNA synthesis by intercalating between base pairs of the DNA/RNA strand, thus preventing the replication of rapidly-growing cancer cells, (2) to inhibit topoisomerase II, preventing the relaxing of supercoiled DNA, and thus blocking DNA transcription and replication, and (3) to create iron-mediated free oxygen radicals that damage the DNA and cell membranes. Anthracyclines-based combination chemotherapy has shown improved anticancer activity than anthracyclines alone. For example, doxorubicin has achieved response rate of 40–50% as single agent while 60–70% in combination [20]. These regimens include doxorubicin or epirubicin with cyclophosphamide (AC and EC); doxorubicin, cyclophosphamide, and fluorouracil (FAC or CAF); epirubicin with cyclophosphamide and fluorouracil (FEC). Unfortunately, the clinical benefits of anthracyclines are limited by cardiotoxicity that can lead to the development of potentially fatal congestive heart failure [21]. The combination of anthracycline and cyclophosphamide (AC) is commonly used as first-line chemotherapy in metastatic breast cancer, with or without fluorouracil. Jassem et al. showed improved response rates of 37% to 57% and median time to progression ranging from 6 to 9 months for fluorouracil + AC-type regimens in phase III trials [22]. These regimens are more active but also more toxic than single agent regimens or nonanthracycline-based combinations [23, 24]. Joensuu et al. reported better response rate of 55% in patients treated with FEC than 48% in patients treated with epirubicin alone. However, most of FEC-treated patients (80%) suffered from total hair loss while majority of epirubicin-treated patients (59%) experienced little or no hair loss. Other chemotherapy-related toxicity were more common in FEC-treated patients including hematologic toxicity, nausea, and vomiting [24]. Furthermore, anthracycline-based regimens have not demonstrated a benefit in overall survival compared to single-agent anthracyclines.

#### 2.1.2. Taxane-Based Regimens

Taxanes are another class of chemotherapy agents originally derived from natural sources then synthetically derivatized including paclitaxel (Taxol) and docetaxel (Taxotere). The mechanism of action of taxanes is to disrupt microtubule function. Microtubules are essential to cell division, and taxanes stabilize GDP-bound tubulin in the microtubule, thereby inhibiting the process of cell division. Therefore taxanes also can be classified as mitotic inhibitors. However due to their poor water-solubility, taxanes encounter difficulties in pharmaceutical formulation and this often results in reduced bioavailability.

Different mechanisms of action of anthracyclines and taxanes provide the rationale of combination therapy of these two classes of drugs. Taxanes and anthracyclines typically do not produce overlapping toxicities with existing therapies. Bria et al. reported improved time to progression and overall survival from doxorubicin with paclitaxel (or docetaxel) therapy compared to anthracycline-based combination therapy (FAC or AC). Although greater hematologic toxicity (such as neutropenia) occurs from taxane containing regimen (74%) than the anthracycline regimen (63%) [18] the overall added toxicity of an anthracycline/taxane combination may be overcome by a substantially greater therapeutic benefit.

Taxane with nonanthracycline combinations is another highly effective regimen and is particularly useful in patients with rapidly progressive visceral metastases, who were previously treated with an anthracycline. In this regimen, capecitabine and gemcitabine are drugs of choices as nonanthracycline drugs for combination with taxanes (docetaxel or paclitaxel). Albain et al. reported the combination of gemcitabine and paclitaxel regimen to be superior to paclitaxel alone with longer time to progression (6 versus 4 months) and better response rate (41% versus 26%). However toxicity of this combination was higher with increased neutropenia (61% versus 22%), fatigue (19% versus 13%), and neuropathy (24% versus 22%) [25].

#### 2.1.3. Other Combination Regimens of Nonspecific Small Molecule Chemotherapeutic Agents

Increased use of anthracyclines and taxanes in adjuvant (given in addition to main treatment) and neoadjuvant (given before the main treatment) settings limits the treatment options for patients upon relapse. Multidrug resistance (MDR) is a major limitation of conventional chemotherapy [26]. This is often a result of overexpression of efflux pump proteins such as P-glycoprotein (P-gp; encoded by MDR1) and multidrug resistance-associated protein (MRP). Some nonanthracycline and nontaxane-containing multidrug regimens have high response rates in MDR tumors. For example, ixabepilone is a nontaxane tubulin polymerizing agent that has low susceptibility to multiple tumor resistance mechanisms. Preclinical data showed that ixabepilone retains activity in tumors that use MDR pumps and in tumors that are paclitaxel-resistant [27]. Ixabepilone in combination with capecitabine (Table 1) results in prolonged progression-free survival relative to capecitabine alone (5.8 versus 4.2 months). Objective response rate was also increased (35% versus 14%). Cyclophosphamide, methotrexate plus fluorouracil (Table 1), is another combination regimen used for treatment of metastatic breast cancer.

As discussed above most combination therapies with small molecule chemotherapeutic agents present improved clinical benefits including enhanced response rate and prolonged overall survival, progression-free survival, relapse-free survival, and/or time to progression. However, with additive efficacy the adverse effects from each agent are compounded resulting in patients' suffering from more treatment-related toxicity. The nonspecific nature of small molecule chemotherapeutics accounts for much of the toxicity due to nonselective biodistribution in healthy tissues concurrently with tumor accumulation. Additionally exposure to multiple conventional chemotherapeutic agents reduces response rate due to increased efflux of these drugs out of the cells mediated by the overexpression of MDR related efflux pumps or transporters [26]. Therefore, the need for reducing treatment-related toxicity and overcoming MDR leads researchers to explore new classes of target-specific anticancer therapy.

### 2.2. Combination of Target-Specific Biologic Agent and Small Molecule Chemotherapeutic Agent

Small molecule chemotherapeutic agents lack cancer cell-specific targeting ability and also affect the fast-dividing normal cells of the body (such as blood cells and the cells lining the mouth, stomach, and intestines). Therefore, the major adverse effects from these chemotherapeutic agents are nonspecific toxicities including anemia, nausea, vomiting, and hair loss. Biologic agents are advantageous to chemotherapy in their ability to actively target-specific receptors. Conventional chemotherapy does not discriminate effectively between tumor cells and rapidly dividing normal cells thus leading to nonspecific adverse effects. In contrast, target-specific anticancer therapies interfere with molecular targets that have an important role in tumor growth or progression distinct from normal cells. Also some of these agents act as inhibitors to MDR-related proteins thereby increasing the response rate [34]. Overall targeted therapies provide a broader therapeutic window with less toxicity and higher response rate compared to conventional chemotherapy. They are often useful in combination with chemotherapy (Figure 1(b)) and/or radiation to produce additive or even synergistic effects with unique mechanism of action than traditional cytotoxic therapy. Target-specific anticancer therapeutic agents can be classified by their structures and mechanism of actions as extracellular targeting monoclonal antibodies and intracellular targeting small molecular tyrosine kinase receptor inhibitors. These agents used in metastatic breast cancer target primarily human epidermal growth factor receptor type 2 (HER2), vascular endothelial growth factor receptor (VEGFR), or epidermal growth factor receptor (EGFR). Currently available target-specific anticancer agent-based combination regimens are listed in Table 2.

#### 2.2.1. Monoclonal Antibody-Based Combination Regimens

Monoclonal antibodies are monospecific antibodies made by identical immune cells as clones of a unique parent cell. Due to their nature monoclonal antibodies can be designed to bind to specific substances hence they are widely used for target specific detection or purification [35]. Approximately 20% of breast cancers overexpress HER2, a transmembrane glycoprotein receptor with tyrosine kinase activity. Overexpression of this receptor is associated with increased disease recurrence and poor prognosis. Trastuzumab (TRZ) is a monoclonal antibody that interferes with the HER2 by several suggested mechanisms of action including (1) inhibit HER2 dimerization, which is essential for further signal transduction (2) reduce available HER2 on the cell surface by endocytosis and (3) introduce antibody-dependent cell-mediated cytotoxicity [36]. The combination of HER2-directed therapy with endocrine therapy is a promising first-line treatment for patients with hormone receptor-positive and HER2-positive metastatic breast cancer that is not imminently life-threatening or symptomatic. For others, the combination of chemotherapy with HER2-targeted therapy in the first line setting is preferred. Several chemotherapeutic agents appear to be synergistic with trastuzumab (TRZ) (Table 2). Robert et al. reported that TRZ plus multiagent combination chemotherapy (e.g., TRZ plus paclitaxel, and carboplatin) improves response rates and progression-free survival, although it also increases toxicity over TRZ plus single-agent chemotherapy.

Bevacizumab, a monoclonal antibody against VEGFR, acts as an inhibitor of angiogenesis. VEGF is an important signaling protein involved in both vasculogenesis (the formation of the circulatory system) and angiogenesis (the growth of blood vessels from preexisting vasculature). Since angiogenesis is the essential way of providing nutrition to tumors and a fundamental step in the transition of tumors from a dormant state to a malignant one, it serves as important target for anticancer therapy. As monotherapy in metastatic breast cancer, it has only modest activity (response rate of 9%) [37]. However, Baselga et al. found that bevacizumab in combination with weekly paclitaxel improves progression-free survival in HER2-negative disease [38].

Cetuximab is a monoclonal antibody that targets overexpressed EGFR in various cancers [39]. EGFR is the cell-surface receptor for members of the epidermal growth factor family. Mutations affecting EGFR expression or activity could result in cancer. EGFR is the most well-known protein overexpressed among triple-negative breast cancer (i.e., lacking expression of the estrogen receptor (ER), progesterone receptor (PR), and HER2 proteins). Although single-agent activity of cetuximab in refractory metastatic breast cancer is limited, cetuximab combined with cisplatin has shown modest activity in patients with triple-negative metastatic breast cancer [38].

Monoclonal antibodies as biologic anticancer agents have shown reduced toxicity while having modest activities. The low response rates due to drug resistance can explain such modest activities. TRZ resistance is developed in about 70% of TRZ-treated breast cancer patients in early treatment period [36] and only small portion of patients (less than 20%) achieved an objective response on cetuximab treatment [40]. The mechanisms of resistance development of monoclonal antibody drugs are not fully understood but mutation on the targeting receptors can explain a part of the mechanisms. To reduce these anticancer drug resistances broader targeting and non-MDR affecting small molecule agents are considered in combination with antibody-based biologics.

#### 2.2.2. Small Molecule Tyrosine Kinase Receptor Inhibitor-Based Combination Regimen

Lapatinib is a small molecule dual tyrosine kinase receptor inhibitor of EGFR and HER2 that, like TRZ, has demonstrated a significant improvement in overall survival when added to the treatment of HER2-positive metastatic breast cancer [53]. The benefit of lapatinib combined with chemotherapeutic agents (Table 2) as compared to chemotherapeutic agents alone was seen in patients with progressive, HER2-overexpressing metastatic breast cancer who were previously treated with an anthracycline, a taxane, and TRZ. Cameron et al. reported that patients treated with combination of lapatinib and capecitabine showed improved overall survival time of 75 weeks compared to that of 64.7 weeks in the patients treated with capecitabine [45]. However due to the broad selectivity of lapatinib, the primary observed toxicities of lapatinib are nonspecific such as diarrhea, acneiform skin rash, nausea, and pruritus [47]. 

Another strategy for targeting VEGF and tumor angiogenesis is the use of small molecule tyrosine kinase receptor inhibitors that target the VEGF receptor (VEGFR), including sunitinib, sorafenib, axitinib, and pazopanib. Gianni et al. reported improved response rate of 72% with the docetaxel plus sunitinib combination compared to 11% with sunitinib monotherapy. Most common side effects of sunitinib are anorexia, fatigue, mucositis, diarrhea, and nausea. However, the combination was well tolerated and did not significantly worsen the toxicity associated with the chemotherapy alone [48]. 

Although these agents, alone or in combination with chemotherapy and/or other biologics, hold great promise, to date they have failed to demonstrate significant activity in metastatic breast cancer [54, 55]. Most small molecule tyrosine kinase receptor inhibitors have dose-related toxicity such as hepatotoxicity compared to monoclonal antibody therapy mainly due to less selective distribution. 

#### 2.2.3. Poly(Adenosine Diphosphate-Ribose) Polymerase Inhibitor-Based Regimen

Poly(adenosine diphosphate-ribose) polymerase (PARP) is a DNA-binding protein involved in detection and repair of DNA strand breaks [56]. PARP inhibitors are a new and exciting class of agents to treat triple-negative and BRCA-mutated breast cancer [57]. Cancers defective in DNA repair, specifically cancers with mutations in the breast cancer associated BRCA1 and BRCA2 genes and triple-negative breast cancer (which shares molecular and pathologic features with BRCA1-related breast cancers) appear to be particularly sensitive to inhibition of PARP-1 [58]. Olaparib (AstraZeneca) is an oral small molecule PARP inhibitor and its clinical evidence of sensitivity towards BRCA-mutated cancers was reported in a study by Gien and Mackay [51]. The early data of ongoing clinical trial by O'Shaughnessy et al. showed promising results of significantly higher response rates (*P* = 0.02) of patients receiving olaparib, gemcitabine, and carboplatin compared to that of placebo and chemotherapy groups [51].

### 2.3. Combination of Target-Specific Biologic Agents

Although not many of the regimens are clinically approved, the concept of combination of two or more target-specific biologic agents is promising (Figure 1(b)). The rationale is to target multiple molecular pathways that lead to the same signaling cascade and hence achieve the synergistic effects. For example when the extracellular domain of HER2 forms a dimer its intracellular tyrosine kinase domain is phosphorylated and downstream signaling cascades are turned on which enhances cancer cell proliferation, prolongation and angiogenesis. By administering a combination of TRZ and lapatinib [52], TRZ can target the extracellular domain of HER2 preventing dimerization while lapatinib can target the intracellular domain for HER2 blocking the phosphorylation. In this case both agents target different parts of the same receptor and hence one can expect the same clinical output [36]. Such dual targeting of HER2 may be synergistic, as suggested by an ongoing clinical trial in metastatic breast cancer patients progressing on one or more prior trastuzumab-containing regimens [59]. The combination therapy resulted in a significant improvement in progression-free survival compared to monotherapy with lapatinib [52]. The combination has also been shown to inhibit HER family receptors more completely than trastuzumab alone and has been effective against trastuzumab resistant tumors [60]. As discussed above each class of target-specific agents still has its own drawbacks such as drug resistance from monoclonal antibodies and nonspecific toxicity and lack of selectivity from small molecule kinase inhibitors.

## 3. Challenges of Currently Used Combination Treatments for Metastatic Breast Cancer

Beneficial therapeutic effectiveness from combination treatment is promising when considering theoretically nonoverlapping mechanisms of action of each anticancer agent. However, current combination treatments in metastatic breast cancer are far from perfect with moderate enhanced efficacy but additive toxicity as described above. Commonly these anticancer agents are administered together as a physical mixture of each agent without pharmacokinetic modification. These agents (free drugs) therefore distribute are eliminated independently of each other. As a result the additive effects are seen not only in anticancer activity but concurrently in adverse effects. Combining molecularly targeted agents is an improved strategy, but brings added complications including patient compliance issue. For example, in HER2 targeted combination therapy with TRZ and lapatinib, these two agents have two different routes of administration. TRZ is given intravenously weekly while lapatinib is administered daily as an oral formulation. Due to two different ways of administration with different schedules it is challenging to manage proper pharmacokinetic and pharmacodynamic profiles and virtually impossible to achieve uniform temporal and spatial codelivery. Storniolo et al. reported the results of a pharmacokinetic study of coadministration of TRZ and lapatinib to 27 patients. Serial blood samples were collected over a 24-hour period after ingestion of the lapatinib dose and/or the initiation of the 0.5-hour TRZ infusion. They reported that lapatinib area under the plasma drug concentration versus time curve within a 24-hour period after dosing and *C*_max⁡_ were not significantly different in comparing the combination with lapatinib alone. AUC_24_ and Cmax of TRZ were not significantly different when comparing the combination to trastuzumab alone [61]. However since the courses of TRZ last almost one year and the possible drug resistance development from chronic tyrosine kinase inhibitor therapy are reported it is not simple to apply this short-term result to chronic combination regimens. Patients would find it difficult to follow the direction which may cause more frequent office visits to improve compliance to the regimen which also increases healthcare costs.

## 4. Current Novel Approaches to Overcome the Challenges: Carrier-Mediated Combination Drug Delivery 

The challenges discussed above have driven researchers to investigate novel approaches by incorporating nanotechnology with combination anticancer treatment. The promising hypothesis is that by delivering two of more drugs simultaneously using a carrier-mediated drug delivery system the combination system can generate synergistic anticancer effects and reduce individual drug related toxicity. However this area of delivering multiple drugs with a single vehicle remains largely unexplored while most research efforts focus on single agent delivery systems. Therefore, here we will review carrier-mediated drug delivery systems containing multiple anticancer agents for cancer treatment in general not limited to metastatic breast cancer. Carrier-mediated drug delivery systems can offer many advantages over delivery of physical mixture of multiple drugs. The advantages include (1) prolonged drug circulation half-life mediated by the carrier, (2) reduced nonspecific uptake, (3) increased accumulation at the tumor site through passive enhanced permeation and retention (EPR) effect and/or active targeting by incorporation of targeting ligands [62], (4) predominantly endocytotic uptake with the potential to bypass mechanisms of multidrug resistance, and (5) ratiometric dosing, that is, ability to tailor the relative ratios of each agent based on its pharmacological disposition. Also a single delivery system carrying multiple drugs in the same platform can lead to synchronized and controlled pharmacokinetics of each drug, resulting in improved drug efficacy, single formulation with improved solubility and bioavailability, and so forth [63]. When carrier-mediated systems containing multiple drugs come to fruition as novel drug delivery systems in general cancer therapy it can also be adapted to metastatic breast cancer treatment, which requires aggressive therapy. Widely investigated carriers for multiple drug delivery such as liposomes, dendrimers, polymeric nanoparticles, and water-soluble polymer-drug conjugates are reviewed below.

### 4.1. Combination Drug Delivery Systems Based on Liposomes

**Figure 2:**
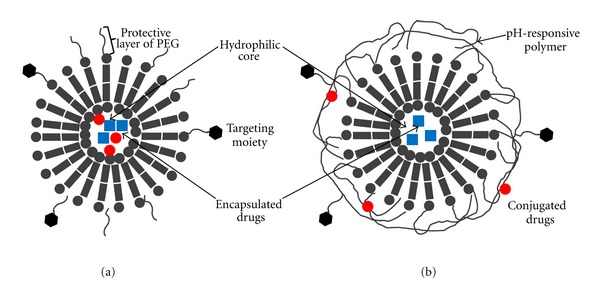
Combination drug delivery systems based on liposomes. (a) Combination of drugs encapsulated in the hydrophilic core of liposome (b) polymer-caged nanobin (PCN); liposome-based hybrid system carrying a combination of encapsulated drug and conjugated drug.

Liposomes are spherical vesicles composed of one or more lipid bilayers with a drug containing aqueous core (Figure 2(a)). Liposomes are one of the most widely used pharmaceutical carriers with several unique characteristics such as (1) ability to encapsulate both hydrophilic and hydrophobic drugs and (2) protecting the encapsulated drugs from the external environment [64]. Unmodified liposomes are rapidly cleared from the blood by phagocytic cells of the reticuloendothelial system (RES), resulting in premature degradation and systemic clearance [64]. To overcome this challenge long-circulating stealth liposomes have been developed by coating the surface with an inert and biocompatible polymer such as polyethylene glycol (PEG). The polymer layer provides a protective shell over the liposome surface and suppresses liposome recognition by opsonins, and therefore prevents rapid clearance by the RES [65]. Several examples of combination drug delivery systems based on liposomes are listed in Table 3. Zucker et al. has developed a PEGylated nanoliposome (LipoViTo) for simultaneous delivery of two chemotherapeutic agents (topotecan and vincristine) [66]. In mice xenograft studies, the simultaneous delivery of two agents by the LipoViTo system altered the biodistribution of each individual drug in favor of the tumor resulting in >100-fold higher tumor levels. This ultimately resulted in a higher therapeutic response (91% tumor suppression) from the dual-drug liposome formulation, which could not be achieved by either administering a combination of free drugs (29% tumor suppression) or liposomal formulations containing only one drug (38–43%). 

Another unique liposomal system is a polymer-caged nanobin (PCN, Figure 2(b)) developed by Lee et al., which illustrates the different ways to incorporate multiple drugs in the same liposome such as encapsulation of one drug and covalent conjugation of the other. PCN comprising of a doxorubicin- (Dox-) loaded liposomal core and surrounded by a cisplatin (Pt) conjugated pH-responsive polymer cage was developed with tunable drug ratios (Pt/Dox) and surface charge potentials. This dual-agent formulation dramatically enhanced the overall efficacy of each drug against breast and ovarian cancer cells at reduced doses. Combination index and isobologram analysis confirmed higher synergistic drug effects over a wider range of concentrations compared to combinations of either the free drugs or nanopackaged single drugs. The extent of synergism was further dependent on the individual drug ratios which highlights the importance of single carrier-mediated combination drug delivery platforms that allow such tunable drug loading. In vitro studies with the PCN system further demonstrated that during cellular uptake via endocytosis, the initial drug-combination ratio in the liposome was preserved [67]. 

Attaching targeting ligands such as antibodies and peptides to a drug carrier is a widely applied strategy drastically increasing carrier accumulation in the desired cells, tissues, and organs. Several such targeted liposomes have been developed for combination drug delivery applications [68]. Wu et al. synthesized and evaluated transferring- (Tf-) conjugated liposomes coloaded with doxorubicin (Dox) and verapamil (Ver). The targeted liposome showed high specificity for Tf receptor overexpressing cancer cells. Due to the weakly basic nature of Dox and Ver, it was possible to load both agents into liposomes via a transmembrane pH gradient. The Dox and Ver coloaded liposome showed threefold increase in anticancer activity compared to liposomal Dox alone while concurrently minimizing Ver-related adverse effects including cardiotoxicity, which typically occur with systemic administration of Ver [69]. In addition, the combination of Tf receptor targeting and coencapsulation of Dox and Ver was highly effective in overcoming MDR in Dox resistant cells. These results indicate that active targeting plays a pivotal role in enhancing receptor-mediated endocytosis of the drug delivery carrier bypassing Pgp-mediated efflux and resistance mechanisms. 

As with any carrier-mediated codelivery system, determination of the optimal dose as the relative ratio of multiple drugs is a complex aspect in liposome-based combination drug delivery system. Mayer et al. reported precise control over combinatorial drug dosing in liposomes [70]. The combination of drugs loaded into liposomes at desirable ratios could be achieved by adjusting liposome synthesis and drug encapsulation process. Various products based on this formulation such as CPX-351 (cytarabine + daunorubicin) [71] and CPX-1 (irinotecan + floxuridine) [72] are currently investigated in clinical trials. 

### 4.2. Combination Drug Delivery Systems Based on Dendrimers

**Figure 3:**
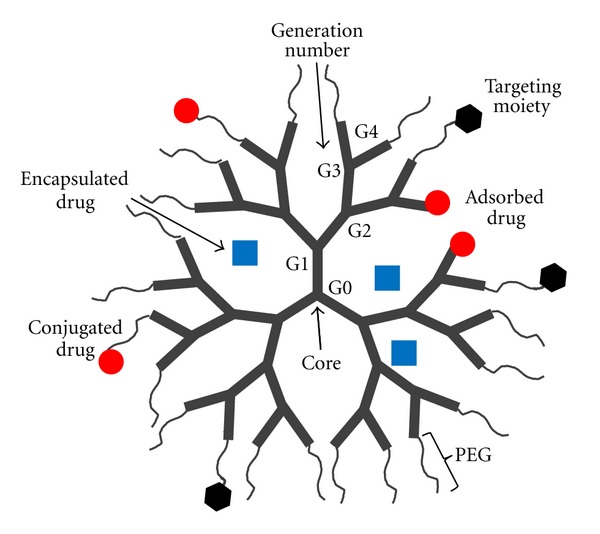
Combination drug delivery systems based on dendrimers: concurrent delivery of water-soluble and -insoluble drugs by adsorption to the surface (ionic interaction), encapsulation within hydrophobic microcavities inside branching clefts or direct covalent conjugation to the surface functional groups.

Dendrimers are well-established three-dimensional, branched polymers that have been thoroughly investigated as controlled and targeted drug delivery systems. The structure of dendrimers can be defined by an initiator core and layers of branched repeating units (each layer is called generations) with functional end groups on the outmost layer (Figure 3). Dendrimers differ from conventional polymers, in that they are nanoscopic in size (1–100 nm), well defined, spherical, possess a high degree of molecular uniformity, and bear ample number of modifiable surface groups [82]. The structural configuration of dendrimers also confers a large drug loading by various techniques such as adsorption to the surface (ionic interaction), encapsulation within hydrophobic microcavities inside branching clefts or direct covalent conjugation to the surface functional groups. These unique properties make dendrimers a desirable platform for concurrent delivery of water-soluble and -insoluble drugs [14, 104]. Examples of dendrimer-based combination drug delivery systems that are currently investigated are listed in Table 4. For example, Ren et al. have developed a poly (amidoamine) (PAMAM) dendrimer for simultaneous co-delivery of gene therapy and chemotherapy agents. 5-fluorouracil (5-FU) was encapsulated in the cavities of the dendrimer core via hydrogen bonding while an antisense microRNA (miR-21) was complexed to the surface through cationic surface charge-based interaction [78]. Successful synchronous delivery of the two therapeutic agents was achieved resulting in synergistic anticancer efficacy, apoptotic activity, and decreased migration ability of the cancer cells compared to each agent alone. In another example Kaneshiro and Lu developed a targeted nanoglobular dendrimer based on a poly(l-lysine) core for intracellular codelivery of doxorubicin (Dox, chemotherapeutic) and siRNA (nucleic acid) [81]. An endothelial targeting peptide c(RGDfK) was conjugated to the dendrimer surface via a PEG spacer. Dox was covalently conjugated while siRNA was complexed to the dendrimer. The targeted dendrimer dual agent delivery system resulted in significantly higher gene silencing efficiency in U87 glioblastoma cells than dendrimer-Dox conjugates or dendrimer siRNA complexes [81]. Lee and coworkers have developed a targetable dendrimer for combination chemoimmunotherapy delivery. A single-stranded DNA-A9 PSMA (prostate-specific membrane antigen) RNA aptamer hybrid was conjugated to a PAMAM dendrimer as the tumor targeting moiety. This system was complexed with a plasmid bearing unmethylated CpG that acts as both an immune-stimulating agent and a carrier of the drug, Dox. The dendrimer-based conjugate showed greater antitumor efficacy with much lower toxicity than the same dose of free Dox or aptamer-free dendrimer conjugate in murine tumor models [79]. 

### 4.3. Combination Drug Delivery Systems Based on Polymeric Nanoparticles

Polymeric nanoparticles are submicron-sized aggregate structures (3–200 nm) that are prepared using random or block copolymers. Polymeric nanoparticles are widely used as drug delivery carriers where the active drug may be physically encapsulated or covalently bound to the polymer matrix depending upon the method of preparation. Several polymeric nanoparticle systems have been explored specifically for combination drug delivery in cancer using both passive and active targeting strategies (Table 5). For example nanoparticles comprising of hydrophobic copolymers such as poly(lactic-co-glycolic acid) (PLGA) [92] and polyalkylcyanoacrylate (PACA) [93] have been used to coencapsulate chemotherapeutic agents and MDR inhibitors for delivery to various cancers. Polymeric nanoparticles can also be formed by self-assembly of amphiphilic block copolymers resulting in a micellar core shell structure. Such a block copolymer typically consists of a hydrophilic or ionic copolymer block and a hydrophobic block that can be a copolymer or a lipid (Table 5). For example, nanomicelles based on diblock copolymers such as PEG/PLGA or PEG/PLA have been used to coencapsulate or conjugate several combinations of anticancer drugs [83–86]. Zhu et al. described a biodegradable cationic nanomicelle based on a triblock copolymer of poly(N,N-dimethylamino-2-ethyl methacrylate)-polycaprolactone-poly(N,N-dimethylamino-2-ethyl methacrylate) (PDMAEMA-PCL-PDMAEMA). The hydrophobic anticancer drug paclitaxel was encapsulated in the micellar core while siRNA was simultaneously complexed to the outer hydrophilic PDMAEMA shell of the micelle [87]. Micellar nanoparticles have also been developed using hybrid block structures such as polymer-lipid blocks for example, PEG-b-[distearoylphosphatidyl ethanolamine] (DSPE) [88, 89], PEG-b-[(cholesteryl oxocarbonylamido ethyl) methyl bis(ethylene) ammonium bromide sebacate] (CES) [90], and PEG-b-[poly(N-hexyl stearate l-aspartamide)] (PEG-b-PHSA) [91]. 

**Figure 4:**
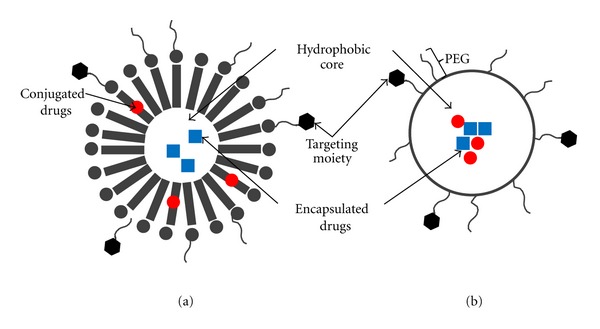
Combination drug delivery systems based on polymeric nanoparticles: (a) micellar polymeric nanoparticle, (b) nonmicellar polymeric nanoparticles.

In general it has been shown that polymeric nanoparticles, compared to liposomes, have greater stability, controlled size distribution, more tunable physicochemical properties, sustained and more controllable drug-release profiles, and higher loading capacity for poorly water-soluble drugs. While majority of the nanoparticle systems described above have demonstrated synergistic therapeutic efficacy in both in vitro and in vivo models some of these studies specifically illustrate that synergistic therapeutic effect is primarily due to the ability to administer two drugs in a tunable mass ratio with predictable spatial and temporal drug release profiles. For example Sengupta et al. developed a hybrid polymeric micelle [88] comprising of a nanoscale PEG-phospholipid block copolymer envelope coating a nuclear PLGA nanoparticle. A chemotherapeutic agent doxorubicin (Dox) was conjugated to the nanoparticle while an anti-angiogenesis agent combretastatin (Com) was trapped within the lipid envelope. The antitumor effect of this tailor made combination drug delivery system was far superior to either physical mixtures of the drugs, mixtures of single agent micellar formulations and even liposomal drug formulations. Detailed biological evaluation showed a good correlation between the spatial-temporal-drug release kinetics and the pathophysiological conditions. It was shown that the disruption of the outer lipid envelope occurred inside a tumor resulting in a rapid deployment of the anti-angiogenesis agent Com, which caused vascular collapse and the intra-tumoral trapping of the nanoparticles. The subsequent slow release of the cytotoxic drug Dox from the nanoparticle killed tumor cells more efficiently by increasing its apoptotic potential (Figure 4). 

### 4.4. Combination Drug Delivery Systems Based on Water-Soluble Polymer Conjugates

**Figure 5:**
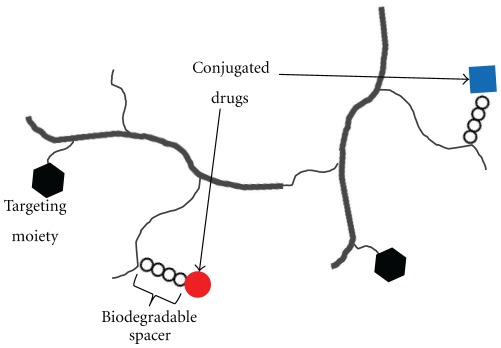
Combination drug delivery systems based on water-soluble polymer conjugates.

Polymer-drug conjugates are drug delivery systems in which a drug is covalently bound to a water-soluble polymeric carrier, normally via a biodegradable linker. Such nanoconstructs were first proposed in the 1970s [105], developed preclinically in the 1980s [106], and started entering the clinical development in the 1990s [107]. Numerous studies are available on water-soluble polymer-drug conjugates including N-(2-hydroxypropyl)methacrylamide (HPMA), PEG, dextran, and polyglutamic acid (PGA) backbones carrying a single drug entity. Only very recently such backbones have been extended to carrying multiple drugs for combination therapy. Polymer conjugates-based combination strategies can be categorized in three groups of (1) polymer-single drug conjugate plus free drug, (2) polymer-single drug conjugate plus polymer-single drug conjugate, and (3) single polymer carrier carrying multiple drugs on the same backbone. Examples of group 1 include coadministration of PGA copolymer-paclitaxel plus platinum based chemotherapeutic agents [108] or radiotherapy [109]. Combinations of HPMA copolymer-Dox conjugate plus HPMA copolymer-phototherapeutic agent conjugate [110] or PEG-ZnPP (heme oxygenase inhibitor) conjugate plus PEG-DAO (enzyme) conjugate [111] are examples of group 2. Examples of group 3 are extremely limited in the literature with only a few drugs being combined within a single polymeric carrier. While groups 1 and 2 have been reviewed elsewhere [112, 113] the preset review is focused on the drug delivery system of combination therapy using a single water soluble polymeric carrier (Figure 5). 

 N-(2-hydroxypropyl)methacrylamide (HPMA) is one example of biocompatible, non-immunogenic, non-toxic water-soluble copolymers that can be tailor-made for specific combination drug delivery needs [114]. The unique characteristics of HPMA copolymers that allow such combination delivery approach feasible include: (1) ability to easily tailor individual drug content in the polymer backbone, (2) covalent linking of drugs to the side chains of polymers via enzymatically or hydrolytically cleavable spacers and (3) ability to vary polymer molecular weight, spacer length and type to systematically control the spatial and temporal release of the drugs. The first conjugate of this type was an HPMA copolymer carrying the combination of endocrine therapy (aromatase inhibitor aminoglutethimide (AGM)) and chemotherapy (Dox), HPMA copolymer-AGM-Dox conjugate [63]. The drug loading in this conjugate was approximately 5% w/w for AGM and 7% w/w for Dox and the drugs were linked via a tetrapeptide linker designed to be cleaved within the lysosomal compartment of cancer cells. In model breast cancer cell lines this polymer dual drugs conjugate was shown to be more active than the combination of two HPMA copolymer conjugates each carrying a single drug. A follow on study suggested that such increased activity could be due to a variety of factors, including drug release rate, conjugate conformation in solution and possibly, activation of certain molecular pathways (induction of apoptosis, e.g., downregulation of Bcl-2 protein) [63, 94]. Generally for a polymer conjugate drug system the biodistribution of the polymer is dependent on its molecular weight, polydispersity, and solution conformation. Hence it is easier to more correctly predict the pharmacokinetics of the individual drugs since they are attached to the same polymer. Another HPMA copolymer conjugate, carrying two chemotherapeutic drugs gemcitabine (Gem) and Dox was developed by Lammers et al. [95] assessed in vivo and proved being able to deliver the two drugs to tumor tissue. HPMA-Gem-Dox was more active and less toxic than the combination of two polymer conjugates each carrying a single drug, and even more than the combination of the free drugs. Furthermore, HPMA-Gem-Dox inhibited angiogenesis and induced apoptosis more strongly than the controls [95]. Segal et al. recently reported an HPMA copolymer containing the antiangiogenic drug TNP-470 and aminobisphosphonate alendronate [97]. Alendronate had the dual function of a bone targeting moiety and a pharmacologically active agent. In vitro this combination conjugate confirmed its antiangiogenic and antitumor properties and in vivo caused complete tumor regression in a human osteosarcoma model [97, 98]. 

Others have explored modifications of the PEG backbone to conjugate a combination of chemotherapeutic agents. While unmodified PEG can only conjugate two drug molecules per chain (one on each end), Pasut et al. developed a PEG with a dendritic structure on one end that allowed coupling of upto 8 nitric oxide (NO) and one epirubicin (EPI) molecule per chain [99, 100]. In vivo studies confirmed that the PEG-NO-EPI conjugate displayed anticancer activity but was less cardiotoxic [100, 101]. This combination is of particular interest as EPI and NO induce different pharmacological responses that are tissue-dependent. In cancer cells, EPI and NO act synergistically, while in cardiomyocytes NO counterbalances EPI induced cardio-toxicity [100]. Conjugation of both drugs onto a single chain ensured that they undergo the same body distribution, thus maximizing the benefits of this combination. A branched PEG polymer was developed by Minko et al. who synthesized a six-branched conjugate containing equimolecular amounts of CPT, BH3, and LHRH. In vitro studies showed that such multidrug-conjugated systems was almost 100 times more cytotoxic than the single conjugates and displayed enhanced antitumor activity in vivo when compared with monotherapy [102]. 

**Figure 6:**
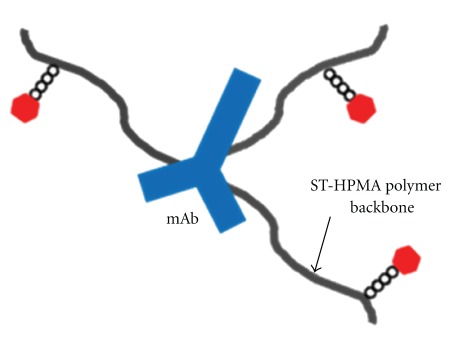
TRZ-STP-PKI166 conjugate.

Our research group has recently proposed a novel carrier-mediated combination drug delivery system for HER2 overexpressing metastatic breast cancer [103]. We synthesized and characterized a star-shaped semitelechelic (ST) HPMA copolymer conjugate containing both TRZ and PKI166 (a small molecule tyrosine kinase inhibitor) covalently linked to the same backbone (Figure 6). The rational is that such a dual drugs conjugate will target and inhibit the extracellular (via TRZ binding) and intracellular (via PKI166 binding) kinase domains of the same HER2 receptors in breast cancer cells. Using a star-like semitelechelic HPMA copolymer structure, an antibody molecule can be conjugated to several ST-HPMA precursors via reactive functional group present only at one end of the polymer chain. This enables single-point attachment to the antibody and results in a well-defined system without cross-linking or branching and narrow molecular weight distribution. ST-HPMA conjugated to TRZ and PKI166 have demonstrated improved stability and bioactivity in HER2 overexpressing breast cancer cell lines. Our results further indicated that the conjugate contained sufficient amount of each agents to produce synergistic anticancer activity. The conjugate drug delivery system was shown to be successfully internalized and localized within HER2 overexpressing breast cancer cells and further prolonged the kinase inhibitory activity of TRZ and PKI166. Polymer conjugated dual drug combination systems such as the one reported could potentially be more effective in vivo due to altered biodistribution mediated by the polymer. The TRZ-STP-PKI166 conjugate therefore appears to be a promising novel drug delivery system that can deliver a combination of drugs with different mechanisms of action for molecularly targeted therapy to overcome the limitations from each individual drug alone (Table 6).

## 5. Conclusions and Future Directions

The presence of two or more therapeutic agents on a single carrier platform offers new therapeutic possibilities but at the same time poses many new challenges. In order to identify an appropriate drug combination, it is necessary to perform thorough biological evaluation which must be supported by a profound understanding of the molecular mechanisms involved. Another critical aspect is the determination of the optimal mass ratio of each component within a combination drug delivery system. This requires systematic research investigating the impact of different drug ratios on the biological activity of the combination delivery systems. Recently a Canadian pharmaceutical company Celator (http://www.celator.ca/) has developed a methodical approach to assess different drug ratios within their liposomal technology resulting in the development of different liposomal formulations that are now being assessed in phase II clinical trials, namely, CPX-1 (irinotecan: floxuridine) and CPX-351 (cytarabine: daunorubicin). Such an approach needs to be extended to other combination delivery systems such as dendrimers or polymer-drug conjugates. Determination of the kinetics of release of each drug in a multidrug combination system will be also necessary to determine the optimum ratio as one drug may affect the release profile of the other drug and thereby affect activity. Finally clinical development of these combination products is extremely challenging, due to developmental costs of designing such complex systems. However, these combination drug delivery system-based therapeutics are likely to be perceived by pharmaceutical companies as novel opportunities to extend the patent lives compared to current blockbuster drugs.

## Figures and Tables

**Table 1:** Clinically used combination regimens of nonspecific small molecule chemotherapeutic agents in metastatic breast cancer.

Classes	Regimens	Advantages	Disadvantages	References
	Doxorubicin + Cyclophosphamide			
Anthracycline based	Doxorubicin + Fluorouracil	Improved RR	No significant difference in time to progression or survival, more treatment related toxicity, and less quality of life	[23, 24]
	Epirubicin + Cyclophosphamide			
	Epirubicin + Fluorouracil			

	Doxorubicin + Paclitaxel	Improved RR and PFS	More hematologic toxicity, cardiotoxicity	[18, 28]
Taxane based	Doxorubicin + Docetaxel			
	Capecitabine + Docetaxel	Improved TTP, RR, and OS	Increased nonhematologic toxicity (diarrhea, stomatitis, hand-foot syndrome)	[25, 29, 30]
	Gemcitabine + Paclitaxel			

Other combinations	Ixabepilone + Capecitabine	Improved RR and TTP in heavily pretreated patient	Peripheral neuropathy	[27]
	Cyclophosphamide + Methotrexate + Fluorouracil	Improved RR, RFS, and OS	Rapid bone loss	[31–33]

OS: overall survival; PFS: progression-free survival; RFS: relapse-free survival; RR: response rate; TTP: time to progression.

**Table 2:** Clinically used combination regimens of target specific biologic agent(s) in metastatic breast cancer.

Classes	Regimens	Advantages	Disadvantages	References
	Trastuzumab + Doxorubicin + Cyclophosphamide	Improved RR, PFS, and OS	Cardiomyopathy, hematologic toxicity	[41, 42]
	Trastuzumab + Epirubicin + Cyclophosphamide			
mAb based	Trastuzumab + other chemotherapy (Paclitaxel, Docetaxel, Vinorelbine, Capecitabine, Platinum compounds, and Gemcitabine)	Improved RR and PFS	Increased hematologic toxicity	[43]
	Bevacizumab + Paclitaxel	Improved PFS	More toxicity (hypertension, proteinuria, bleeding, nasal septum perforation, thromboembolic event, heart failure, mortality)	[44]
	Cetuximab + Cisplatin	Improved RR and PFS in patients with TNBC	More acne-like rash, neutropenia, dyspnea	[38]

	Lapatinib + Capecitabine			
	Lapatinib + Paclitaxel	Improved RR, TTP, PFS	More toxicity (toxicity from chemotherapy plus diarrhea, skin rash, nausea, pruritis)	[45–47]
Tyrosine kinase inhibitor based	Lapatinib + Letrozole			
	Sunitinib + Docetaxel	No worsen toxicity	Nonsignificant combination activity	[48]
	Erotinib + Cisplatin + Gemcitabine	Well tolerated	No survival benefit	[49]

PARP inhibitor based	Iniparib + Gemcitabine + Carboplatin	Improved PFS and OS	Neutropenia, thrombocytopenia, anemia, fatigue or asthenia, leukopenia	[50]
	Olaparib + Gemcitabine + Carboplatin	Improved RR		[51]

Multiple targeted	Trastuzumab + Lapatinib	Improved PFS and Overcome TRZ resistance	Additive toxicity from TRZ and Lapatinib, patient compliance issue (IV and oral administration)	[52]

OS: overall survival; PFS: progression-free survival; RFS: relapse-free survival; RR: response rate; TTP: time to progression; TRZ: trastuzumab.

**Table 3:** Combination drug delivery systems based on liposomes.

Formulation	Therapeutics	Indication	Status	Targeting	References
PEG-Liposome	Topotecan + Vincristine	Brain cancer	In vivo	Passive	
Polymer-caged nanobins (PCN); Liposome surrounded by cholesterol-terminated poly(acrylic acid)	Cisplatin + Doxorubicin	Various cancers	In vitro	Passive	[67]
Liposome	Cytarabine + Daunorubicin	Acute myeloid leukemia	Phase II	Passive	[71]
Liposome	Irinotecan + Floxuridine	Colorectal cancer	Phase II	Passive	[72, 73]
Mixture of two Liposomes	Irinotecan + Cisplatin	Small-cell lung cancer	In vivo	Passive	[74]
PEG-Liposome	Quercetin + Vincristine	Hormone- and TRZ-insensitive breast cancer	In vivo	Passive	[75]
Cationic, anionic PEG-Liposome	siRNA + Doxorubicin	MDR-breast cancer	In vivo	Passive	[76]
Liposome	6-Mercaptopurine + Daunorubicin	Acute myeloid leukemia	In vitro	Passive	[77]
Transferrin- (Tf-) conjugated PEG-Liposome	Doxorubicin + Verapamil	MDR-leukemia	In vitro	Active (Tf receptor)	[69]

PEG: polyethylene glycol; MDR: multidrug resistant; TRZ: trastuzumab.

**Table 4:** Combination drug delivery systems based on dendrimers.

Carrier composition	Therapeutics	Indication	Status	Targeting	References
G5 PAMAM dendrimer	Antisense-miRNA21 + 5-FU	Glioblastoma	In vitro	Active; miRNA overexpression	[78]
Aptamer-G4 PAMAM dendrimer conjugates	Unmethylated CpG-ONTs + Doxorubicin	Prostate cancer	In vivo	Active; a single-strand DNA-A9 PSMA, RNA aptamer hybrid	[79]
Dendritic PEG	Paclitaxel + alendronate	Cancer bone metastases	In vivo	Active; Bone metastasis	[80]
RGDfK-G3 Poly-lysine dendrimer	Doxorubicin + siRNA	Glioblastoma	In vitro	Active; *α*_*v*_ *β*_3_ integrin	[81]
Folate-G5 poly-propyleneimine dendrimer with ethylenediamine core	Methotrexate + all-trans-retinoic acid	Leukemia	In vitro	Active; folate receptor	[82]

PAMAM: poly (amidoamine); PEG: polyethylene glycol; PSMA: prostate-specific membrane antigen; ONT: oligonucleotides; 5-FU: 5-fluorouracil.

**Table 5:** Combination drug delivery systems based on polymeric nanoparticles.

Carrier composition	Therapeutics	Indication	Status	Targeting	References
*Polymer-polymer micellar nanoparticles*					
PEG-PLGA	Lonidamine + Paclitaxel	MDR breast cancer	In vivo	Active; EGFR	[83]
Methoxy PEG-PLGA	Doxorubicin + Paclitaxel	Various cancer	In vitro	Passive	[84]
PEG-PLA	Paclitaxel, Etoposide, or Docetaxel + 17-AAG	Various cancer	In vitro	Active; HSP90	[85]
PEG-PLA	Combretastatin A4 + Doxorubicin	Various cancer	In vitro	Active; angiogenesis	[86]
PDMAEMA-PCL-PDMAEMA	Paclitaxel + siRNA	Prostate cancer	In vitro	Active; VEGF	[87]

*Polymer-Lipid micellar nanoparticles*					
PEG-DSPE/PLGA	Combretastatin + Doxorubicin	Lung carcinoma	In vivo	Passive	[88]
PEG-PLA and PEG-DSPE/TPGS	Paclitaxel + 17-AAG (HSP90 inhibitor)	Ovarian cancer	In vitro	Active; HSP90	[89]
P(MDS-co-CES)	Paclitaxel + Interleukin-12 or siRNA	Breast cancer	In vivo	Active; Bcl-2	[90]
PEG-b-PHSA	Doxorubicin + Wortmannin	Breast cancer	In vitro	Passive	[91]

*Nonmicellar polymeric nanoparticles*					
PLGA	Vincristine + Verapamil	Hepatocellular carcinoma	In vitro	Passive	[92]
PACA	Doxorubicin + Cyclosporine A	Various cancer	In vitro	Passive	[93]

17-AAG: 17-allylamino-17-demethyoxygeldanamycin; EGFR: epidermal growth factor receptor; HSP90: heat shock protein; PDMAEMA-PCL-PDMAEMA: poly(N,N-dimethylamino-2-ethyl methacrylate)-polycaprolactone-poly(N,N-dimethylamino-2-ethyl methacrylate); PEG: polyethylene glycol; PEG-b-PHSA: PEG-block-poly(N-hexyl stearate l-aspartamide); PEG-PLA: polyethylene glycol-poly lactic acid; PEG-DSPE: PEG-distearoylphosphatidyl ethanolamine; PACA: polyalkylcyanoacrylate; PLGA: poly(d,l-lactide-co-glycolide); P(MDS-co-CES): poly(N-methyldietheneamine sebacate)-co-[(cholesteryl oxocarbonylamido ethyl) methyl bis(ethylene) ammonium bromide] sebacate; TPGS: tocopheryl polyethylene glycol; VEGF: vasculature epidermal growth factor.

**Table 6:** Combination drug delivery systems based on water-soluble polymer conjugates.

Carrier composition	Therapeutics	Indication	Status	Targeting	References
HPMA copolymer	Aminoglutethimide + Doxorubicin	Breast cancer	In vitro	Passive	[63, 94]
HPMA copolymer	Gemcitabine + Doxorubicin	Prostate cancer	In vivo	Passive	[95]
HPMA copolymer	Doxorubicin + Dexamethasone	General cancer	In vivo	Passive	[96]
HPMA copolymer	TNP-470 + Alendronate	Bone metastasis	In vivo	Active; bone	[97]
HPMA copolymer	Paclitaxel + Alendronate	Bone metastasis	In vivo	Active; bone	[98]
Branched PEG	Epirubicin + Nitric oxide		In vivo	Passive	[99–101]
Branched PEG	Camptothecin + BH3 domain peptide		Iv vivo	Active; LHRH	[102]
HPMA copolymer	Trastuzumab + PKI166	HER2 overexpressed breast cancer	In vitro	Active; HER2	[103]

HER2: human epidermal growth factor receptor type 2; HPMA: N-(2-hydroxypropyl)methacrylamide; LHRH: luteinizing-hormone release hormone.
